# Comparison of neurofilament light and heavy chain in spinal muscular atrophy and amyotrophic lateral sclerosis: A pilot study

**DOI:** 10.1002/brb3.2997

**Published:** 2023-04-17

**Authors:** Jin‐Yue Li, Yi Dai, Xiao‐Han Sun, Hai‐tao Ren, Dong‐chao Shen, Xun‐Zhe Yang, Ming‐Sheng Liu, Li‐Ying Cui

**Affiliations:** ^1^ Department of Neurology Peking Union Medical College Hospital, Chinese Academy of Medical Science & Peking Union Medical College Beijing China; ^2^ Department of Neurology Xiamen Branch, Zhongshan Hospital, Fudan University Xiamen China; ^3^ Neuroscience Center Chinese Academy of Medical Sciences Beijing China

**Keywords:** amyotrophic lateral sclerosis, motor neuron disease, neurofilament, spinal muscular atrophy

## Abstract

**Background:**

Spinal muscular atrophy (SMA) and amyotrophic lateral sclerosis (ALS) were two major motor neuron diseases with similar symptoms and poor outcomes. This study aimed to identify potential biomarkers in disease monitoring and differential diagnosis of adult SMA patients with sporadic ALS patients.

**Methods:**

This was a pilot study with ten adult SMA patients and ten ALS patients consecutively enrolled during hospitalization. Serum and cerebrospinal fluid (CSF) samples were collected for assessment of neurofilament light (NFL) and phosphorylated neurofilament heavy chain (pNFH). Serum creatine kinase (CK) and creatinine (Cr) were also compared between groups. The receiver operating characteristic (ROC) curves were used to identify differentiated values among ALS and SMA patients.

**Results:**

Serum Cr, CSF NFL, and CSF pNFH levels of ALS patients were significantly higher than those of the adult SMA patients (*p* < .01). Serum CK and Cr were strongly correlated with baseline ALSFRS‐R scores in SMA patients (*p* < .001). The ROC curves revealed an area under the curve (AUC) of 0.94 in serum Cr with a cut‐off value of 44.5 μmol/L (Sensitivity 90%, Specificity 90%). AUC from the ROC curve of CSF NFL and CSF pNFH were 1.0 and 0.84, with cut‐off values of 1275 pg/mL and 0.395 ng/mL, respectively (Sensitivity and Specificity of 100% and 100% in CSF NFL; Sensitivity and Specificity of 90% and 80% in CSF pNFH).

**Conclusion:**

CSF NFL and pNFH might be useful biomarkers for differential diagnosis of adult SMA and ALS.

## Graphical abstract

Spinal muscular atrophy (SMA) and amyotrophic lateral sclerosis (ALS) are two distinct motoneuron diseases with similar progressive symptoms and severe disability. Neurofilament light (NFL) and phosphorylated neurofilament heavy chain (pNFH) in cerebrospinal fluid might be useful biomarkers for differential diagnosis of SMA and ALS.

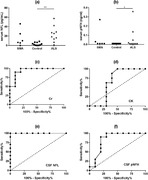

## 1 BACKGROUND

Motor neuron diseases represent a group of specific disorders with the involvement of upper and/or lower motor neurons, resulting in muscle weakness and atrophy (Foster & Salajegheh, 2019). Amyotrophic lateral sclerosis (ALS) is the most common motor neuron disease with unknown etiology and limited treatment options (Oskarsson et al., 2018). Most ALS patients presented with gradually progressed symptoms and eventually died from respiratory failure in 3–5 years (Oskarsson et al., 2018). Similarly, other motor neuron diseases such as spinobulbar muscular atrophy (SBMA) and spinal muscular atrophy (SMA), also progressively developed dysarthria and limb weakness without efficient therapeutic methods clinically for a long time (Arnold & Merry, 2019; Ross & Kwon, 2019). With the development of medical technology, new and promising treatments for motor neuron diseases emerged, such as the most representative disease‐modifying therapy in SMA patients (Mercuri et al., 2020). Although several disease‐modifying treatments including nusinersen and onasemnogene abeparvovec have been therapeutically validated in multiple clinical studies, early diagnosis and treatment are critical to therapeutic efficacy in SMA patients (Schorling et al., 2020). The diagnosis of SMA in patients with symptom onset in childhood or adolescence may be easy to identify, but it is more confusing for late‐onset SMA patients, especially when compared with sporadic ALS patients with symptoms of predominant involvement of lower motor neurons. Useful and reliable biomarkers for early diagnosis and therapeutic monitoring of SMA have recently gained wide attention and were studied by many researchers.

Given the pathological degeneration of motor neurons in motor neuron diseases, disruption and release of cytoskeleton structures may provide specific biomarkers for early diagnosis and disease monitoring. Neurofilament proteins are the major component of neurons with crucial functions in maintaining structure, signaling and transcription, and axonal transport (Didonna & Opal, 2019; Yuan et al., 2017). Previous studies have explored the role of neurofilaments (NFs) in neurological disorders including Alzheimer's disease (de Wolf et al., 2020), Parkinson's disease (Mollenhauer et al., 2020), and multiple sclerosis (Kuhle et al., 2019), and found its correlation with disease severity and progression. Thus, this pilot study aimed to address the potential role of NFs in the evaluation of disease severity in motor neuron diseases, and investigate the diagnostic utility of neurofilament in distinguishing SMA patients from ALS patients, which might give clues about the pathological features that the two disease share.

## 2 METHODS

### 2.1 Participants

This is a pilot study consecutively enrolled patients hospitalized in the Peking Union Medical College Hospital from October 2019 to April 2022. Diagnosis of SMA was performed according to the genetic diagnostic criteria of homozygous or hemizygous deletion in exon 7 or/and exon 8 of the *SMN1* gene. The ALS patients fulfilled the revised El Escorial criteria of clinically definite, probable, or laboratory‐supported probable ALS with predominant involvement of lower motor neurons. The involvement of upper motor neurons (total scores of UMN were 0–16) was measured by clinical signs including increased or clonic tendon reflexes, spasticity, loss of superficial abdominal reflexes, pseudobulbar features, clonic jaw jerk gag reflex, exaggerated snout reflex, forced yawning, Hoffmann reflex and extensor plantar response. All participants were older than 18 years and completed the lumbar puncture during hospitalization. This study was approved by the ethics committees of Clinical Research of Peking Union Medical College Hospital (Beijing, China), and all participants provided informed consent.

Demographic and clinical features were collected from all participants. The revised ALS Function Rating Scale (ALSFRS‐R) was used to evaluate the neurological function of patients. The disease progression rate (DPR) was the monthly decline rate of ALSFRS‐R scores. Pulmonary function was measured in all SMA patients, and the results of forced vital capacity (FVC) were recorded at enrollment. Concurrently, serum creatine kinase (CK) and creatinine (Cr) were measured at the laboratory departments of Peking Union Medical College Hospital by the enzymatic method, as well as the routine CSF parameters including white cell count (WBC) and total protein.

### 2.2 Sample collection and measurement

Blood and CSF samples were collected during the first admission. Serum aliquots were obtained after being centrifuged (3000 × *g* for 10 min) of within 2 h of blood collection, which were stored with CSF samples at −80°C for further analysis. Additionally, serum samples were taken from ten healthy volunteers for comparison of biomarkers. Serum neurofilament light (NFL) concentrations were analyzed by single molecular array (AstraBio, Suzhou, China) assay and the fully automated instrument AST‐DX90 Analyzer (AstraBio, Suzhou, China) following the manufacturer's instructions. The mean interassay and intraassay CV were both less than 10%. We measured the concentrations of NFL in CSF using the commercially available enzyme‐linked immunosorbent assay (ELISA) kits (IBL, Hamburg, Germany), and the mean intraassay coefficient of variation (CV) was less than 20%. For measurements of phosphorylated neurofilament heavy chain (pNFH) concentrations, another ELISA kit (EUROIMMUN AG, Lübeck, Germany) was used with an intraassay of less than 5%. All the measurements were performed by research assistants who were blinded to the diagnosis and clinical data.

### 2.3 Statistical analysis

Statistics were carried out using SPSS 22.0, and graphs were drawn with Graphpad Prism 7. Values of NFL and pNFH that are below the lower limit of detection (LLOD) were approximated to the lowest concentration of detection (0.01 pg/mL of NFL, 0.01 ng/mL of pNFH), and those that exceed the higher limit of measurement were approximated to the highest concentration of detection (10,000 pg/mL of NFL, 10 ng/mL of pNFH). Numerical variables are expressed as mean ± standard deviation or median (interquartile range), which were compared with two‐tailed *t*‐tests or Mann–Whitney test. Categorical variables were described as numbers and percentages and were compared with Pearson's chi‐squared test in groups. Pearson's chi‐squared test was used for proportion values. Group comparisons of CK, Cr, NFL, pNFH, and CSF routine parameters at baseline were performed with the Mann–Whitney test or Kruskal–Wallis *H* test. Group comparisons of ALSFRS‐R scores were performed with Student's *t*‐test. Correlations between variables were assessed with nonparametric Spearman's correlation analyses. Diagnostic values of serum and CSF parameters between SMA patients and ALS patients were visualized by receiver operating characteristic (ROC) curves, and the optimal cut‐off was calculated by the highest Youden Index. *p* Values < .05 were considered significant.

## 3 RESULTS

### 3.1 Clinical characteristics

A total of 20 patients completed the study. Table 1 summarizes the demographic characteristics, clinical features, and laboratory data of SMA patients, ALS patients, and normal controls. Eight male and two female patients diagnosed with SMA were enrolled in this study with copies of the *SMN2* gene ranging from 2 to 4; of those, two were SMA type 2 and eight were SMA type 3. The mean age during hospitalization was 26.80 ± 6.37 years old (ranging from 18 to 38 years old) in SMA patients, which was significantly younger than patients with ALS and normal controls (*p* < .001). The median disease duration from disease onset to sampling was 14 months (IQR: 7–29 months) in ALS patients, which was significantly lower than disease duration in SMA patitents (*p* < .001). Only one patient with ALS had bulbar onset, while all patients had spinal involvement. The mean UMN scores were 5.9 points in ALS patients. No significant difference in sex was found between SMA patients, ALS patients, and normal controls. Motor functions evaluated by ALSFRS‐R were not significantly different between SMA patients and ALS patients (*p* > .05). However, the DPR was significantly higher in ALS patients than in SMA patients (*p* < .001). The FVC was lower than 80% in seven patients with SMA, and the median FVC was 56.95 % (IQR: 36.93‐85.68 %).

**TABLE 1:** The demographic, clinical and laboratory characteristics of SMA, ALS, and control

	SMA (*n* = 10)	ALS (*n* = 10)	Control (*n* = 10)	*p* Value	*p*’ Value	*P*” Value
Age (years)	26.80 ± 6.37	50.00 ± 11.32	46.40 ± 11.18	**<.00** **1**	**<.001**	.483
Sex (male)	8(80%)	6 (60%)	7 (70%)	.329	.606	.639
Disease duration (months)	275.0 (138.0, 343.3)	14.0 (7.0, 29.0)		**<.001**		
ALSFRS‐R	32.60 ± 10.28	38.40 ± 5.97		.144		
DPR	0.06 (0.03, 0.09)	0.47 (0.16, 1.93)		**<.001**		
**Laboratory parameters**						
Serum CK (U/L)	91.0 (55.3, 811.0)	257.0 (110.3, 329.3)		.266		
Serum Cr (μmol/L)	22.5 (11.0, 36.5)	56.50 (45.75, 67.75)		**.001**		
Serum NFL (pg/mL)	8.19 (4.03, 30.18)	22.44 (12.50, 32.72)	2.35 (0.23, 6.49)	.193	.092	**.001**
Serum pNFH (ng/mL)	0.01 (0.01, 0.09)	0.01 (0.01, 0.07)	0.01 (0.01, 0.01)	.803	.059	**.031**
CSF WBC	0.5 (0, 2.5)	2.0 (0.8, 2.5)		.251		
CSF Pro (g/L)	0.32 (0.24, 0.44)	0.42 (0.38, 0.69)		.086		
CSF NFL (pg/mL)	596.97 (395.26, 699.70)	6582.05 (2657.83, 10000.00)		**<.001**		
CSF pNFH (ng/mL)	0.20 (0.18, 0.24)	2.07 (0.46, 3.75)		**.009**		

*Note*: Continuous data are given as mean ± SD or median (IQR) as appropriate. *p* represent the value of comparison between ALS and SMA; *p*’ represent the value of comparison between SMA and normal control; “*p*” represent the value of comparison between ALS and normal control.*P*‐value<0.0 is shown in bold.

ALSFRS‐R, revised ALS functional rating scale; DPR, disease progression rate; CK, creatine kinase; Cr, creatinine; CSF, cerebrospinal fluid; NFL, neurofilament light chain; pNFH, phosphorylated neurofilament heavy chain.

### 3.2 Relationship between biomarkers with clinical features

The age at enrollment was not correlated with ALSFRS‐R scores or any laboratory parameters in SMA and ALS patients, but the onset age was positively correlated with the CK and Cr levels in SMA patients and CSF total protein in ALS patients (*p* < .05, Supplementary Tables S1 and S2). The ALSFRS‐R scores were also strongly correlated with the serum levels of CK and Cr in SMA patients (*p* < .001, *r* = 0.927; *p* < .001, *r* = 0.924, respectively), while significant correlation of ALSFRS‐R scores in ALS patients was only found with serum pNFH levels (*p* = .015, *r* = −0.735). The rate of disease progression was inversely correlated with Cr levels in SMA patients, while no significant correlation between DPR and biomarkers was found in ALS patients. For the pulmonary function in SMA patients (Supplementary Table S1), we found FVC was positively correlated with onset age and ALSFRS‐R scores (*p* < .01), and inversely correlated with DPR (*p* < .01). Moreover, the CK and Cr levels were also positively correlated with FVC in SMA patients.

For the correlation between different laboratory parameters (Supplementary Table S2), we found the NFL levels in CSF were significantly correlated with pNFH levels in CSF among ALS patients (*p* = .005, *r* = 0.804), while no correlation was found between NFL and pNFH levels in serum (*p* > .05). We also did not find a correlation between NFL and pNFH levels both in serum and CSF among SMA patients (*p* > .05). In addition, the NFL levels in serum were strongly correlated with NFL levels in CSF among ALS patients (*p* = .006, *r* = 0.791). However, no correlation between NFL levels in serum and CSF was found in SMA patients (*p* > .05).

### 3.3 Comparisons of laboratory parameters

**FIGURE 1:**
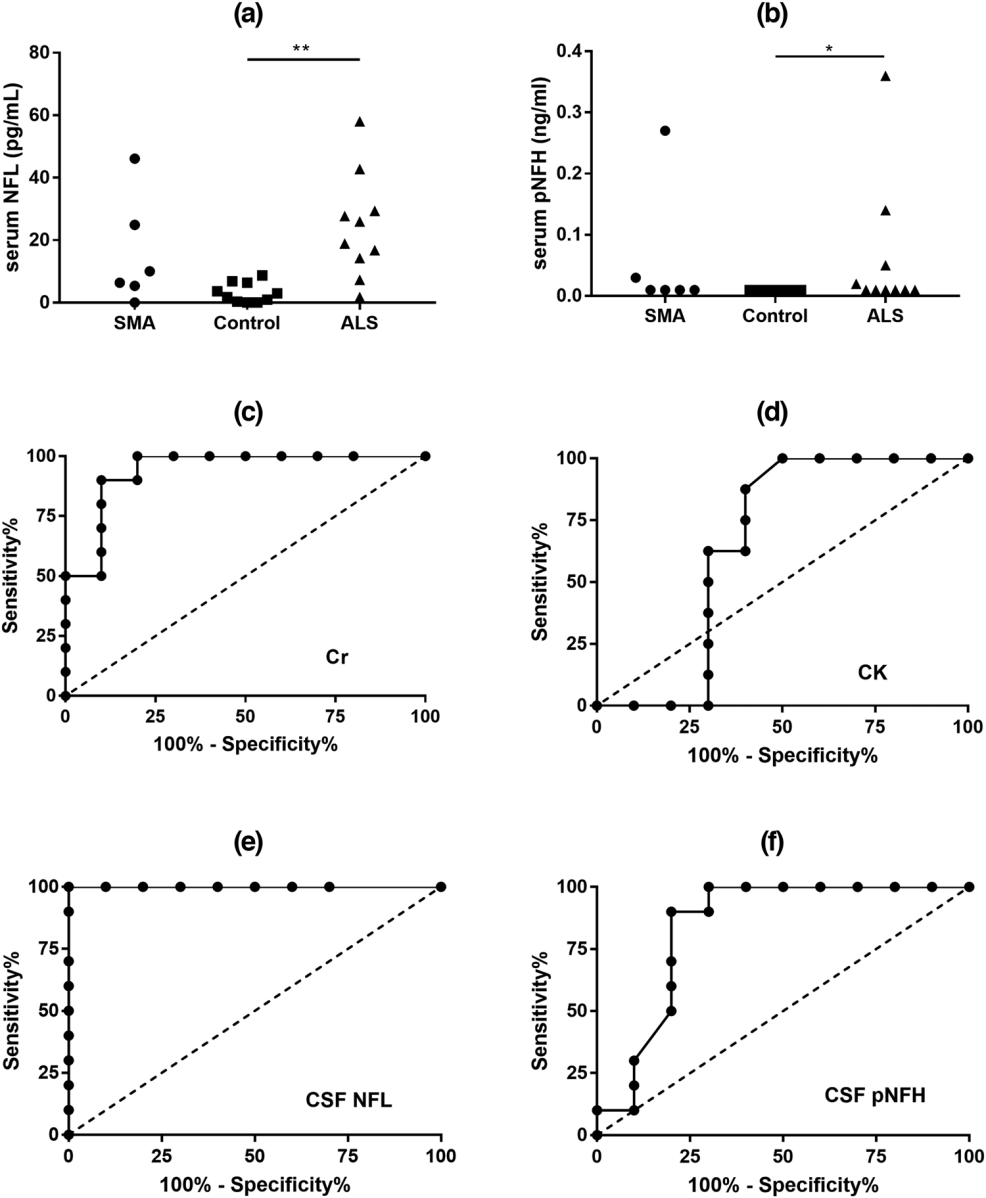
Comparisons of serum levels of NFL and pNFH, and receiver operating curves (ROC) for classifying SMA and ALS. Serum levels of NFL (a) and pNFH (b) were compared between SMA patients, ALS patients, and normal controls. ROC curves of Cr (c), CK (d), CSF NFL (e), and CSF pNFH (f) were generated for discriminating between SMA and ALS patients. *p < .05, **p < .01. CK, creatine kinase; Cr, creatinine; CSF, cerebrospinal fluid; NFL, neurofilament light chain; pNFH, phosphorylated neurofilament heavy chain.

We compared laboratory parameters among different groups (Table 1). The serum Cr levels were significantly higher in ALS patients than those in SMA patients (*p* < .001), while no significant difference in serum CK levels was found between ALS patients and SMA patients (*p* > .05). The serum NFL and pNFH levels in ALS patients were significantly higher than those in normal controls (*p* < .05, Figure 1A and B). However, there was no significant difference in serum NFL and pNFH levels between the SMA and controls, as well as between SMA and ALS patients (Figure 1A and B). It should be noted that the value of pNFH was lower than LLOD in all the controls (*n* = 10) and most patients (ALS: *n* = 6; SMA: *n* = 4), and those values below the LLOD were approximated to the lowest concentration of detection (0.01 ng/mL).

For all CSF samples from patients and controls, WBC was within the normal range (0–8) and did not differ in groups (Table 1). CSF total protein was slightly higher in ALS patients than in SMA patients (*p* = .086). Both CSF NFL and pNFH levels were significantly different between SMA patients and ALS patients (*p* < .01). The CSF levels of NFL in ALS patients were significantly higher than in SMA patients (*p* < .001), and similarly, the CSF pNFH levels in ALS patients were significantly higher than in SMA patients (*p* < .01).

Then, we tested whether these biomarkers could differentiate SMA patients from ALS patients using the ROC curves. Serum Cr showed a significant value of differentiating SMA patients from ALS patients and the cut‐off value was > 44.5 μmol/L (Sensitivity = 90%, Specificity = 90%, AUC = 0.94, *p* < .001, Figure 1C), while serum CK did not (Figure 1D). Serum NFL and pNFH also did not show significant value in the differential diagnosis of SMA and ALS patients. The AUC of CSF NFL was 1 with a cut‐off value of < 1275 pg/mL in the differential diagnosis of two groups (Sensitivity = 100%, Specificity = 100%, *p* < .001, Figure 1E). We also observed that CSF pNFH < 0.395 ng/mL could differentiate SMA patients from ALS patients with 90% sensitivity and 80% specificity (AUC = 0.84, *p* = .01, Figure 1F).

## 4 DISCUSSION

Traditionally, SMA and ALS are two distinct motoneuron diseases with progressive symptoms and severe disability. With the emergence of gene therapies, SMA patients achieve promising improvements in motor function and lifespan (Panagiotou et al., 2022; Waldrop et al., 2020). Moreover, early diagnosis and treatment are critical to therapeutic efficacy in SMA patients (Kong et al., 2021). In clinical practice, however, it is often difficult to distinguish adult SMA patients and ALS patients with the predominant involvement of lower motor neurons by clinical symptoms or electromyography. This pilot study compared the levels of NFs between SMA and ALS patients, and found significantly lower CSF levels of NFL and pNFH in SMA patients than in ALS patients, indicating the potential diagnostic value of NFs in motoneuron diseases. The distinct differences in NFs levels may result from different pathology and patterns of motor neuron degeneration. The main pathogenesis mechanisms of SMA were reduced levels of the survival of motor neuron (SMN) protein resulting from mutation of the *SMN1* gene, which was essential for the maintenance of cellular homeostasis in motor neurons (Lefebvre & Sarret, 2020). It has been suggested that SMN protein would be located in the dendrites and axons of motor neurons during development and played an essential role in correct axonal growth (Chaytow et al., 2018). Thus, SMN protein deficiency leads to disruption of normal cytoskeleton development resulting in shorter axons in SMA patients, which may partly explain the lower NFs levels in SMA patients than in ALS patients. Moreover, the timepoints and disease duration of neuromuscular disruption and motor neuron death were different in the two diseases (Comley et al., 2016; Nash et al., 2016). Autopsy and animal studies identified active degeneration of motor neuron axons that began during embryogenesis and kept progressing in infancy in SMA (Kong et al., 2021), which were different from ALS with symptoms that occurred later for some unknown reason.

Inconsistent with results from the CSF cohort, serum NFL and pNFH were not significantly different among SMA and ALS patients in this pilot study. Moreover, we found a significant association between serum and CSF levels of NFL in ALS patients, which is in line with previous reports (Benatar et al., 2020), but a similar association did not show in SMA patients. Wurster et al. (2020) also did not find a relationship between serum and CSF NFL levels in a cohort of adolescent and adult SMA patients. In another study performed by De Wel et al. (2022), serum NFL was not correlated with CSF NFL whereas serum pNFH was correlated with CSF pNFH in adult SMA patients. However, studies on children with SMA suggested that NFL levels in serum were strongly correlated with NFL levels in CSF (Nitz et al., 2021). The possible explanation for these controversial findings could be the different extents of blood‐brain barrier dysfunction in SMA patients with various types and duration. It is also likely that the circulating clearance of NFL probably differs in different ages of patients and also differs from clearance of pNFH. Further studies are needed to identify the possible value of serum NFL and pNFH in diagnosis and distinguishing SMA from ALS.

Our preliminary results also showed that serum levels of pNFH were negatively associated with ALSFRS‐R scores in ALS patients, suggesting that pNFH may be a biomarker for disease severity. However, no correlation between NFs and motor function was found in SMA patients. Contrary to our findings, previous studies (Darras et al., 2019; Wurster et al., 2020) reported higher serum pNFH and NFL levels in SMA patients with more severe motor function. Differences in the selection of study subjects and instruments for motor function measurement may contribute to inconsistent results. In the present study, we enrolled adult patients with SMA type 2–3 rather than infants/children patients, extending the knowledge of different pathological axonal degeneration and metabolism in older SMA patients.

The widely available biomarkers, CK and Cr, have been increasingly reported for diagnosis and prognostic prediction in neurodegenerative diseases (Ceccanti et al., 2020; Lombardi et al., 2019). In this pilot study, we also found significantly lower Cr in SMA patients than in ALS patients, and higher CK and Cr were strongly associated with higher ALSFRS‐R scores. These findings are consistent with previous observations (Freigang et al., 2021; Milella et al., 2021), suggesting that CK and Cr are potential markers of disease severity in SMA. No association of NFs was found with CK or Cr in SMA patients, these findings indirectly corroborated the hypothesis that degeneration of motor neurons and muscle was independently involved in the pathogenesis of SMA (Habets et al., 2022; Kim et al., 2020). The underlying mechanism is still unknown, and further studies are needed to explore the possible link.

This pilot study had the major advantage of enrollment with both ALS and SMA patients, and comparing the serum and CSF levels of NFs among different groups. But we included a relatively small sample size due to the rarity of SMA, which limited the accuracy and reliability of our results. Besides, age was significantly lower in SMA patients than in ALS patients and controls, and the differences may affect the levels of NFs. Although our results did not show a significant correlation of age with serum or CSF levels of NFL and pNFH, some previous studies noted significantly higher levels of NFL in younger children (Nitz et al., 2021). Thus, we are planning to do further studies with larger sample sizes and matched controls.

## 5 CONCLUSION

In this pilot study, our findings indicated the diagnostic performance of CSF NFL and pNFH in differentiating adult SMA and sporadic ALS patients, expanding the available data on the potential biomarkers for motor neuron diseases. Until now, the diagnosis of SMA is based on molecular testing for homozygous deletion or mutation of the *SMN1* gene, and it is recognized as first line of investigation due to its highly efficiency and reliable results. Our biomarker study indicated its potential role in diagnostic and disease monitoring role, probably providing new information for understanding the pathways leading to neurodegeneration in motor neuron diseases. Future larger case‐control studies with different types of patients are required to validate the values of these biomarkers in patients with motor neuron diseases, increasing our understanding of pathophysiologic differences between SMA and ALS.

## CONFLICT OF INTEREST STATEMENT

The authors report there are no competing interests to declare.

## FUNDING INFORMATION

The CAMS Innovation Fund for Medical Sciences, Grant/Award Number: 2021‐I2M‐1‐003; Strategic Priority Research Program of the Chinese Academy of Sciences “Biological basis of aging and therapeutic strategies”, Grant/Award Number: XDB39040100; Chinese Academy of Medical Science Neuroscience Center Fund “Molecular diagnosis and pathogenesis of ALS”, Grant/Award Number: 2014xh0601_A322102; Chinese Academy of Medical Science Neuroscience Center Fund “Molecular diagnosis and neural network of ALS”, Grant/Award Number: 20141001_A322104.

## Supporting information


**Supplementary Table S1**. Correlation between clinical features and biomarkers in SMA patients.
**Supplementary Table S2**. Correlation between clinical features and biomarkers in ALS patients.

## Data Availability

The data sets used or analyzed during the current study are available from the corresponding author on reasonable request.
