# Supplementary material for: Comparison of neurofilament light and heavy chain in spinal muscular atrophy and amyotrophic lateral sclerosis: A pilot study
Source: Brain Behav. 2023 Apr 17;13(5):e2997. doi: 10.1002/brb3.2997 (PMC10175988; doi:10.1002/brb3.2997)
Supplement: Supplementary file 1 — Supplementary Table S1. Correlation between clinical features and biomarkers in SMA patients. Supplementary Table S2. Correlation between clinical features and biomarkers in ALS patients. [file BRB3-13-e2997-s001.docx]

**Supplementary Table 1.** Correlation between clinical features and biomarkers in SMA patients

| SMA | Onset age | Duration | ALSFRS-R | DPR | CK | Cr | S-NFL | S-NFH | C-Pro | C-NFL | C-NFH |
| --- | --- | --- | --- | --- | --- | --- | --- | --- | --- | --- | --- |
| Duration | p=0.062, r=-0.608 | / | p=0.024, r=-0.701 | p=0.446, r=0.273 | p=0.006, r=-0.794 | p=0.004, r=-0.815 | p=0.544, r=-0.314 | p=0.899, r=0.068 | p=0.686, r=0.147 | p=0.578, r=0.201 | p=0.603, r=0.188 |
| ALSFRS-R | p<0.001, r=0.905 | p=0.024, r=-0.701 | / | p=0.004, r=-0.811 | p<0.001, r=0.927 | p<0.001, r=0.924 | p=0.787, r=-0.143 | p=0.604, r=0.27 | p=0.741, r=0.12 | p=0.576, r=-0.202 | p=0.841, r=-0.073 |
| DPR | p=0.001, r=-0.869 | p=0.446, r=0.273 | p=0.004, r=-0.811 | / | p=0.082, r=-0.576 | p=0.03, r=-0.681 | p=0.156, r=0.657 | p=0.949, r=0.034 | p=0.541, r=-0.22 | p=0.763, r=0.109 | p=0.881, r=0.055 |
| CK | p=0.012, r=0.754 | p=0.006, r=-0.794 | p<0.001, r=0.927 | p=0.082, r=-0.576 | / | p<0.001, r=0.936 | p=0.787, r=0.143 | p=0.512, r=0.338 | p=0.588, r=0.196 | p=0.555, r=-0.213 | p=0.58, r=-0.2 |
| Cr | p=0.001, r=0.884 | p=0.004, r=-0.815 | p<0.001, r=0.924 | p=0.03, r=-0.681 | p<0.001, r=0.936 | / | p=0.957, r=-0.029 | p=0.848, r=0.101 | p=0.647, r=0.166 | p=0.907, r=-0.043 | p=0.626, r=-0.176 |
| S-NFL | p=0.397, r=-0.429 | p=0.544, r=-0.314 | p=0.787, r=-0.143 | p=0.156, r=0.657 | p=0.787, r=0.143 | p=0.957, r=-0.029 | / | p=0.512, r=0.338 | p=0.538, r=-0.319 | p=0.577, r=-0.29 | p=0.787, r=-0.143 |
| S-NFH | p=0.949, r=0.034 | p=0.899, r=0.068 | p=0.604, r=0.27 | p=0.949, r=0.034 | p=0.512, r=0.338 | p=0.848, r=0.101 | p=0.512, r=0.338 | / | p=0.949, r=0.034 | p=0.146, r=0.669 | p=0.069, r=0.778 |
| C-Pro | p=0.86, r=0.064 | p=0.686, r=0.147 | p=0.741, r=0.12 | p=0.541, r=-0.22 | p=0.588, r=0.196 | p=0.647, r=0.166 | p=0.538, r=-0.319 | p=0.949, r=0.034 | / | p=0.54, r=-0.221 | p=0.661, r=-0.159 |
| C-NFL | p=0.75, r=0.116 | p=0.578, r=0.201 | p=0.576, r=-0.202 | p=0.763, r=0.109 | p=0.555, r=-0.213 | p=0.907, r=-0.043 | p=0.577, r=-0.29 | p=0.146, r=0.669 | p=0.54, r=-0.221 | / | p=0.132, r=0.511 |
| C-NFH | p=0.763, r=0.109 | p=0.603, r=0.188 | p=0.841, r=-0.073 | p=0.881, r=0.055 | p=0.58, r=-0.2 | p=0.626, r=-0.176 | p=0.787, r=-0.143 | p=0.069, r=0.778 | p=0.661, r=-0.159 | p=0.132, r=0.511 | / |
| FVC | p=0.001, r=0.875 | p=0.067, r=-0.6 | p<0.001, r=0.945 | p=0.006, r=-0.794 | p=0.001, r=0.879 | p=0.001, r=0.888 | p=0.787, r=-0.143 | p=0.604, r=0.27 | p=0.736, r=0.122 | p=0.828, r=-0.079 | p=0.855, r=-0.067 |

ALSFRS-R, revised ALS functional rating scale; DPR, disease progression rate; CK, creatine kinase; Cr, creatinine; S, serum; C, cerebrospinal fluid; Pro, total protein; NFL, neurofilament light chain; pNFH, phosphorylated neurofilament heavy chain; FVC, forced vital capacity.

**Supplementary Table 2.** Correlation between clinical features and biomarkers in ALS patients

| ALS | Onset age | Duration | ALSFRS-R | DPR | CK | Cr | S-NFL | S-NFH | C-Pro | C-NFL | C-NFH |
| --- | --- | --- | --- | --- | --- | --- | --- | --- | --- | --- | --- |
| Duration | p=0.557, r=0.212 | / | p=0.088, r=0.566 | p=0.001, r=-0.887 | p=0.69, r=0.169 | p=0.054, r=0.624 | p=0.051, r=-0.63 | p=0.319, r=-0.351 | p=0.387, r=0.307 | p=0.103, r=-0.545 | p=0.134, r=-0.508 |
| ALSFRS-R | p=0.867, r=0.061 | p=0.088, r=0.566 | / | p=0.005, r=-0.805 | p=0.776, r=0.12 | p=0.531, r=0.226 | p=0.432, r=-0.28 | p=0.015, r=-0.735 | p=0.657, r=-0.161 | p=0.198, r=-0.444 | p=0.086, r=-0.643 |
| DPR | p=0.763, r=-0.109 | p=0.001, r=-0.887 | p=0.005, r=-0.805 | / | p=0.42, r=-0.333 | p=0.138, r=-0.503 | p=0.09, r=0.564 | p=0.052, r=0.628 | p=0.761, r=-0.111 | p=0.056, r=0.62 | p=0.117, r=0.527 |
| CK | p=0.399, r=-0.347 | p=0.69, r=0.169 | p=0.776, r=0.12 | p=0.42, r=-0.333 | / | p=0.955, r=-0.024 | p=0.16, r=-0.548 | p=0.577, r=-0.234 | p=0.307, r=-0.415 | p=0.002, r=-0.898 | p=0.086, r=-0.643 |
| Cr | p=0.65, r=0.164 | p=0.054, r=0.624 | p=0.531, r=0.226 | p=0.138, r=-0.503 | p=0.955, r=-0.024 | / | p=0.385, r=-0.309 | p=0.457, r=-0.266 | p=0.229, r=0.419 | p=0.88, r=-0.055 | p=0.803, r=-0.091 |
| S-NFL | p=0.275, r=0.383 | p=0.051, r=-0.63 | p=0.432, r=-0.28 | p=0.09, r=0.564 | p=0.16, r=-0.548 | p=0.385, r=-0.309 | / | p=0.102, r=0.546 | p=0.574, r=0.203 | p=0.006, r=0.791 | p=0.029, r=0.685 |
| S-NFH | p=0.283, r=0.377 | p=0.319, r=-0.351 | p=0.015, r=-0.735 | p=0.052, r=0.628 | p=0.577, r=-0.234 | p=0.457, r=-0.266 | p=0.102, r=0.546 | / | p=0.268, r=0.388 | p=0.2, r=0.442 | p=0.285, r=0.376 |
| C-Pro | p=0.017, r=0.728 | p=0.387, r=0.307 | p=0.657, r=-0.161 | p=0.761, r=-0.111 | p=0.307, r=-0.415 | p=0.229, r=0.419 | p=0.574, r=0.203 | p=0.268, r=0.388 | / | p=0.287, r=0.374 | p=0.622, r=0.178 |
| C-NFL | p=0.46, r=0.265 | p=0.103, r=-0.545 | p=0.198, r=-0.444 | p=0.056, r=0.62 | p=0.002, r=-0.898 | p=0.88, r=-0.055 | p=0.006, r=0.791 | p=0.2, r=0.442 | p=0.287, r=0.374 | / | p=0.005, r=0.804 |
| C-NFH | p=0.51, r=0.237 | p=0.134, r=-0.508 | p=0.086, r=-0.643 | p=0.117, r=0.527 | p=0.086, r=-0.643 | p=0.803, r=-0.091 | p=0.029, r=0.685 | p=0.285, r=0.376 | p=0.622, r=0.178 | p=0.005, r=0.804 | / |

ALSFRS-R, revised ALS functional rating scale; DPR, disease progression rate; CK, creatine kinase; Cr, creatinine; S, serum; C, cerebrospinal fluid; Pro, total protein; NFL, neurofilament light chain; pNFH, phosphorylated neurofilament heavy chain.
